# Visualizing the Invisible: Advanced Optical Microscopy as a Tool to Measure Biomechanical Forces

**DOI:** 10.3389/fcell.2021.706126

**Published:** 2021-09-06

**Authors:** Chad M. Hobson, Jesse S. Aaron, John M. Heddleston, Teng-Leong Chew

**Affiliations:** ^1^Advanced Imaging Center, Janelia Research Campus, Howard Hughes Medical Institute, Ashburn, VA, United States; ^2^Cleveland Clinic Florida Research and Innovation Center, Port St. Lucie, FL, United States

**Keywords:** biomechanical force, fluorescence microscopy, mechanobiology, light-sheet fluorescence microscopy, super-resolution microscopy

## Abstract

The importance of mechanical force in biology is evident across diverse length scales, ranging from tissue morphogenesis during embryo development to mechanotransduction across single adhesion proteins at the cell surface. Consequently, many force measurement techniques rely on optical microscopy to measure forces being applied by cells on their environment, to visualize specimen deformations due to external forces, or even to directly apply a physical perturbation to the sample via photoablation or optogenetic tools. Recent developments in advanced microscopy offer improved approaches to enhance spatiotemporal resolution, imaging depth, and sample viability. These advances can be coupled with already existing force measurement methods to improve sensitivity, duration and speed, amongst other parameters. However, gaining access to advanced microscopy instrumentation and the expertise necessary to extract meaningful insights from these techniques is an unavoidable hurdle. In this Live Cell Imaging special issue Review, we survey common microscopy-based force measurement techniques and examine how they can be bolstered by emerging microscopy methods. We further explore challenges related to the accompanying data analysis in biomechanical studies and discuss the various resources available to tackle the global issue of technology dissemination, an important avenue for biologists to gain access to pre-commercial instruments that can be leveraged for biomechanical studies.

## Introduction

Mechanical forces play important roles in many aspects of biology. They are known to modulate homeostasis (Guillot and Lecuit, 2013), intracellular signaling pathways (Liu et al., 1999; Han et al., 2004; Thompson et al., 2012), gene expression (Goldspink et al., 1992; Tajik et al., 2016), cell-cell interaction (Basu et al., 2016; Angulo-Urarte et al., 2020; Markovič et al., 2020), cancer progression (Kumar and Weaver, 2009; Jain et al., 2014), cardiovascular functions (Bishop and Lindahl, 1999; Pesce and Santoro, 2017; Beech and Kalli, 2019), and development (Mammoto and Ingber, 2010; Agarwal and Zaidel-Bar, 2021). Yet, studies of biomechanical force can be rife with unique challenges (Roca-Cusachs et al., 2017). Specifically, (i) mechanical forces themselves cannot be directly labeled for visualization as with other biological components, (ii) the magnitude of many biological forces necessitates exquisite sensitivity for accurate quantification, (iii) integrating force measurement assays with live cell microscopy is often a complex engineering challenge, (iv) measuring forces can unwittingly perturb the biological systems or processes being studied, and (v) the complexity of subsequent data analysis can often hinder interpretation of results.

Assays that quantify biomechanical forces are often the result of interdisciplinary work that combines framing a biological hypothesis, synthesizing a force-sensing substrate or sensor, designing or adapting imaging instrumentation or other readout mechanisms, and careful analysis to extract meaningful information from the data. Many of the currently available force measurement tools have previously been expertly discussed (Bao and Suresh, 2003; Polacheck and Chen, 2016; Roca-Cusachs et al., 2017). Here, we will provide a brief synopsis of these force measurement methods as an overview. However, one of the most notable commonalities of many force measurement assays is their reliance on optical instrumentation to visualize and quantify cellular mechanical force. As a result, the choice of optical instrumentation matters immensely in determining the accuracy and sensitivity of the experimental readout. In light of this, our Review focuses on the constraints that microscopy places on many mechanobiological techniques, and how emerging advanced microscopy methods can be leveraged to overcome some of these limitations. Additionally, we will offer a guided tour of how readers can access this cadre of instruments, some of which have yet to be commercialized. Unfortunately, the hurdles facing biologists do not end with restricted access to emerging imaging technologies. The data size and complexity produced by modern microscopes can be daunting (Ouyang and Zimmer, 2017). We discuss some of the considerations that should be made when handling and analyzing this deluge of data. We will conclude by offering a perspective of remaining challenges, and how they may provide important opportunities for future development.

## Force Measurement Techniques

**FIGURE 1:**
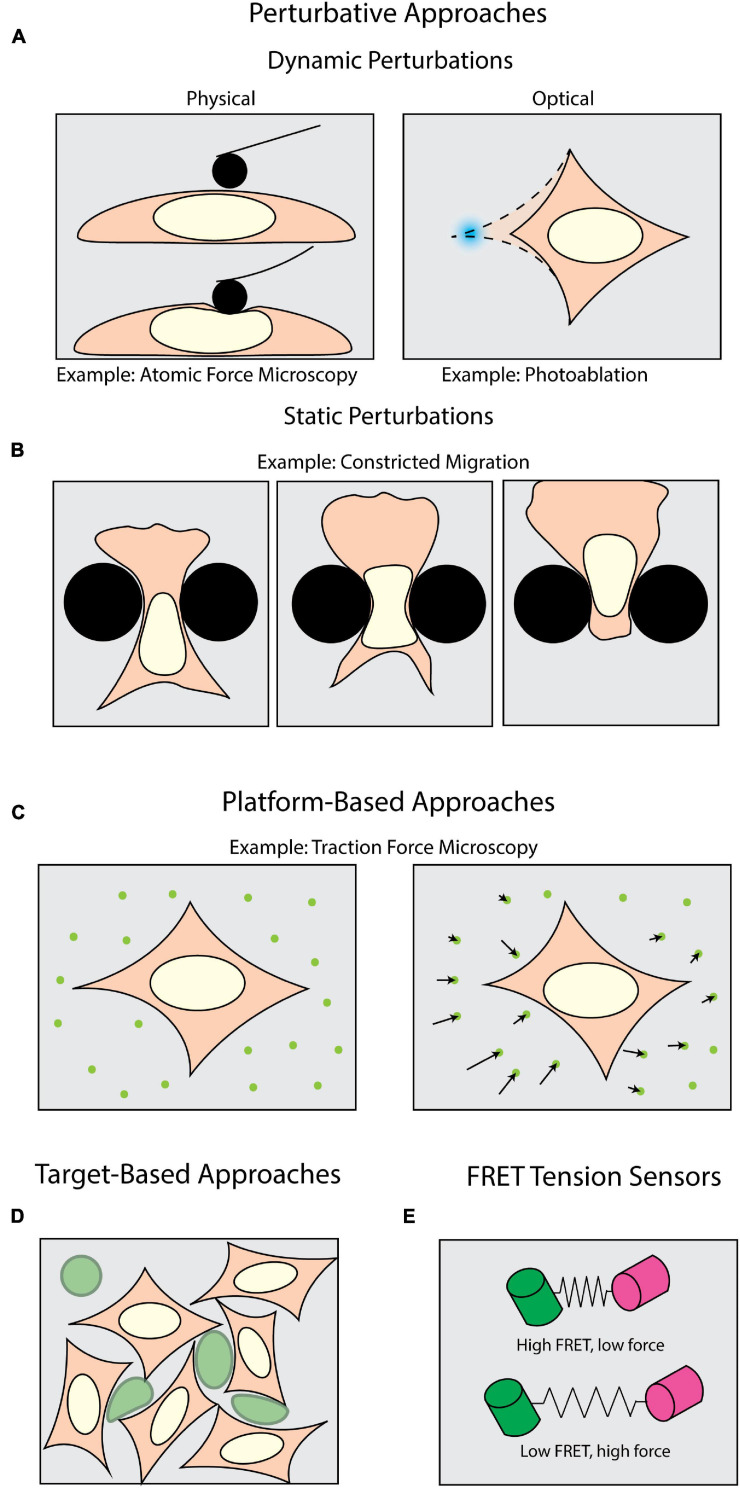
Methods of measuring forces in biology. Perturbative techniques can be either **(A)** dynamic or **(B)** static. **(A)** Dynamic methods such as atomic force microscopy (AFM) use external physical probes to measure the force required to deform the sample. Dynamic optical perturbations use either photoablation to reveal underlying tension (as shown) or optogenetic tools to activate force generation. **(B)** Static perturbations (e.g., constricted migration assays) use rigid physical barriers to induce large-scale shape changes as cells navigate their environment. **(C)** Platform-based approaches, such as Traction Force Microscopy (TFM), monitor displacements of fiducial markers to measure mechanical forces cells apply to their surroundings. **(D)** Target-based approaches (e.g., liquid droplets) measure the deformation of micron-scale particles (green) to infer forces generated within tissues or during target engagement. **(E)** FRET tension sensors use molecular springs between donor and acceptor fluorophores to convert FRET signals to intracellular forces.

The proper function of biological systems requires the intricate coordination between biochemical and mechanical signaling. Together, these signals allow living systems to respond to external and internal cues that span a wide range of biological length scales (Ingber, 2003; Discher et al., 2005; Guillot and Lecuit, 2013; Cho et al., 2017; Agarwal and Zaidel-Bar, 2021; Evers et al., 2021). Unlike many biochemical readouts, biomechanical forces must be measured *in situ*, in context, and transiently. Furthermore, the magnitude of these forces spans a large dynamic range (Du Roure et al., 2005; Sun et al., 2005; Rauzi et al., 2008; Xia et al., 2018). Yet, mechanical forces cannot be directly visualized, and are usually dependent on innovative methods to infer and quantify their location, direction, and magnitude. While some of these mechanobiology methods are capable of directly measuring small magnitude forces, most methods require light microscopy for visualization and quantification (Polacheck and Chen, 2016; Roca-Cusachs et al., 2017). Conversely, microscopy can also act as a limiting factor for the precision and sensitivity of force measurement methods. Here, we will survey commonly used force measurement techniques (Figure 1) with an emphasis on how their implementations are dependent upon fluorescence microscopy.

A plethora of tools have been developed to apply and measure forces at the cellular and sub-cellular level. One class of tools can be broadly described as perturbative – that is, the sample is actively deformed by some external means (Figure 1A). Physically perturbative techniques can be further categorized into whether the method can measure forces without light microscopy. Techniques such as atomic force microscopy (Krieg et al., 2019) and magnetic tweezers (Gosse and Croquette, 2002) directly measure the magnitude of the dynamically applied force without fluorescence microscopy. However, they can also be coupled with light microscopy to add additional dimensionality to an experiment (Beicker et al., 2018; Nelsen et al., 2020). Other physically perturbative techniques, such as micropipette aspiration (Hochmuth, 2000), substrate stretching (Caille et al., 1998; Moraes et al., 2010), and single-pipette micromanipulation (Neelam et al., 2015) necessitate fluorescence imaging to visualize the deformation of a sample. This supports the quantification of intracellular strains, stresses, and viscoelastic properties (Wu et al., 2018; Krieg et al., 2019). These dynamic methods are well-suited for replicating scenarios wherein samples experience perturbative forces from their environment. However, these capture only a subset of the conditions in which a cell may be subject to an external force.

To introduce additional molecular specificity, the illumination source in most modern microscopes can be leveraged to optically induce *in situ* perturbations through light-mediated potentiation of molecular motors (Figure 1A) with high spatiotemporal resolution. This is usually achieved with various optogenetic tools (Airan et al., 2009; Wu et al., 2009; Fenno et al., 2011; Oakes et al., 2017). These techniques also allow the experimenter to subsequently perform rapid live-cell observation. For example, photoactivatable Rac1 has been developed to induce and study cell motility, protrusions, and ruffling (Wu et al., 2009). It is also important to note that the maintenance of force (isometric tension) can play an equally important biomechanical role as transient force generation. Tension is a steady-state phenomenon, and even though it can be measured across larger platforms such as cell monolayers (Kolodney and Wysolmerski, 1992; Goeckeler and Wysolmerski, 1995), the *in situ* tension will be more effectively visualized upon its disruption. One means of targeting this disruption is through photoablation, which uses high-intensity focused light to break molecular bonds (Müller et al., 1991; Vogel and Venugopalan, 2003; Vogel et al., 2005). Subsequent observation of the relaxation area surrounding the ablation then allows one to infer the tension and forces prior to disruption.

Another interesting consideration is that under physiological conditions, perturbative forces can be exerted both dynamically and statically. In the case of cells undergoing significant shape changes to navigate through tight interstitial spaces, the cells are subjected to self-generated forces against static barriers (Figure 1B; Paul et al., 2017). One means of mimicking such forces is the use of microfabricated substrates with micron-scale features and chemoattractant gradients (Heuzé et al., 2011; Davidson et al., 2015; Paul et al., 2016). Among many things, this class of force measurement techniques has historically been useful in measuring how cells dynamically respond to static perturbations (McGregor et al., 2016; Paul et al., 2017). Physically perturbative techniques, both static and dynamic, can leverage microscopy to visualize the deformations being induced upon a sample, but do not give insight into the magnitudes of and means by which cells apply forces to their surroundings. Accomplishing this necessitates a shift from visualizing the deformation of the sample to the deformation of the environment.

Measuring the force cells exert on their surroundings requires an experimental milieu that contains fluorescent fiducial markers and is deformable by cell-scale forces. These “platform-based” approaches primarily make use of optical tracking and computational modeling to monitor deformations induced by the biological samples and extract biomechanical forces (Figure 1C). Traction force microscopy (TFM) is one of the most well-established, platform-based methods, originating from the observation that migrating cells can deform thin, elastic substrates (Harris et al., 1980; Dembo and Wang, 1999). These methods rely on embedding fluorescent beads in a substrate upon which cells migrate (Munevar et al., 2001a,b). Forces exerted on the substrate lead to translation of the fiducial markers, which is then computationally converted into force vectors (Han et al., 2015). TFM can be used not only to map forces in two (2D) and three dimensions (3D) (Franck et al., 2011; del Álamo et al., 2013; Legant et al., 2013; Toyjanova et al., 2014), but also as a function of time (Peschetola et al., 2013). Another commonly used platform-based approach is micropillar arrays. Conceptually related to TFM, these assays use microscopy to track the bending of flexible micropatterned pillars (Tan et al., 2003; Xiao et al., 2018), which is then converted to force information through computational modeling (Schoen et al., 2010). This can be expanded for use with cell monolayers (Saez et al., 2010). Furthermore, magnetic actuation of post arrays can facilitate simultaneous force application and measurement in this type of assay (Sniadecki et al., 2007; Monticelli et al., 2018). Collectively these assays are tailored for investigating force generation (Shiu et al., 2004; Du Roure et al., 2005; Jannat et al., 2011; Umeshima et al., 2019). However, biomechanical forces occur under a multitude of other, more complex physiological conditions, thus necessitating additional methods beyond platform-based approaches.

In measuring intercellular forces within complex tissue or whole organisms, many investigators have turned toward “target-based” approaches (Figure 1D). The premise of target-based methods is to measure the shape change of an introduced object with a known stiffness. This has been achieved with liquid microdroplets or micron-scale polyacrylamide spheres, enabling investigators to study phenomena such as tissue morphogenesis (Campàs et al., 2014; Serwane et al., 2017; Mongera et al., 2018; Träber et al., 2019; Hofemeier et al., 2021), interstitial pressure in tumor growth (Dolega et al., 2017; Lee et al., 2019), and phagocytosis (Vorselen et al., 2020, 2021). However, none of the methods discussed so far directly identify the molecular source from which a force is potentiated. Accomplishing this requires introducing a genetically encoded mechanical sensor.

One of the most sensitive types of intracellular force sensors utilizes Förster Resonance Energy Transfer (FRET). FRET is a process by which energy from one fluorophore (the donor) is transferred to a neighboring fluorophore (the acceptor), typically when they are less than 10 nm apart (Jares-Erijman and Jovin, 2003). This allows for precise quantification of proximity, which can be leveraged to quantify mechanical forces (LaCroix et al., 2015; Gayrard and Borghi, 2016). Such “FRET tensions sensors” (Meng and Sachs, 2012; Cost et al., 2019) consist of a donor and acceptor fluorophore joined by a linker capable of sensing intramolecular tension within a molecule of interest (Figure 1E; LaCroix et al., 2015; Gayrard and Borghi, 2016). By calibrating the molecular spring stiffness, one can sensitively measure changes in force with single pN sensitivity (Freikamp et al., 2017; Ringer et al., 2017). For comparison, the detection range of TFM spans 100s of pN to 10s of nN (Style et al., 2014). FRET sensors have been of particular interest for studying tension across focal adhesions and their associated proteins (Grashoff et al., 2010; Ringer et al., 2017).

One important message from the technical survey above is that the accuracy and sensitivity of many of these assays is dependent on the capacity of the microscope to deliver the appropriate readout. In fact, the optical detection step is often the ultimate limitation of a force measurement assay. Further problems can often arise if microscopy instruments are not chosen carefully or the most appropriate instrument is not available. The optimal integration of light microscopy into a mechanobiological assay requires an equally detailed understanding of the microscope performance. In the following sections, we will discuss the major imaging parameters to consider when performing force measurements, and how advanced microscopy methods can be leveraged to improve them in complex biological systems.

## Integrating Advanced Light Microscopy With Force Measurements

Fluorescence microscopy is fundamentally a game of trade-offs between several key imaging parameters, such as speed, dimensions, resolution, and phototoxic effects. The optimal balance is usually determined by both the quantitative experimental question as well as the characteristics of the sample. No single microscopy method is ideally suited to balance these imaging parameters for all specimens. Since the effects of biomechanical forces can manifest in countless biological processes, a broad diversity of samples – ranging from single cells to developing embryos – necessitates unique microscopy techniques. In this Review, we detail some of the imaging parameters critical for force measurements, and how the new generation of microscopes can tackle these previously unattainable parameters. In addition, we provide case studies wherein well-considered use of microscopy is beneficial to mechanobiological studies.

### Resolution

Biology encapsulates a broad range of length scales, from individual molecules spanning mere nanometers to whole embryos measuring millimeters in length. Any method for visualizing biological processes, however, is subject to the physics of light – diffraction places a fundamental limitation on the minimum distance at which two objects can be distinguished, known as resolution. Resolution is a function of both the microscope numerical aperture (NA) and the wavelength of the emitted light (Amos et al., 2012; Goodwin, 2014), and is conventionally limited to a few hundred nanometers. As a consequence, the final, acquired image is not a true representation of the object being imaged. Due to light diffraction, the image is blurred by (i.e., convolved with) the point spread function (PSF) of the microscope (Pawley, 2006). Furthermore, most microscopes do not offer isotropic resolution, with the axial resolution being more severely compromised (Amos et al., 2012). When using microscopy to measure biological forces, any limitations in resolution will constrain the sensitivity and accuracy of force measurements. Therefore, in studying forces occurring on particularly small length scales, such as those across individual focal adhesions during cell migration (Beningo et al., 2001), the resolution of the microscope must be appropriately matched to the scale of the forces of interest.

**FIGURE 2:**
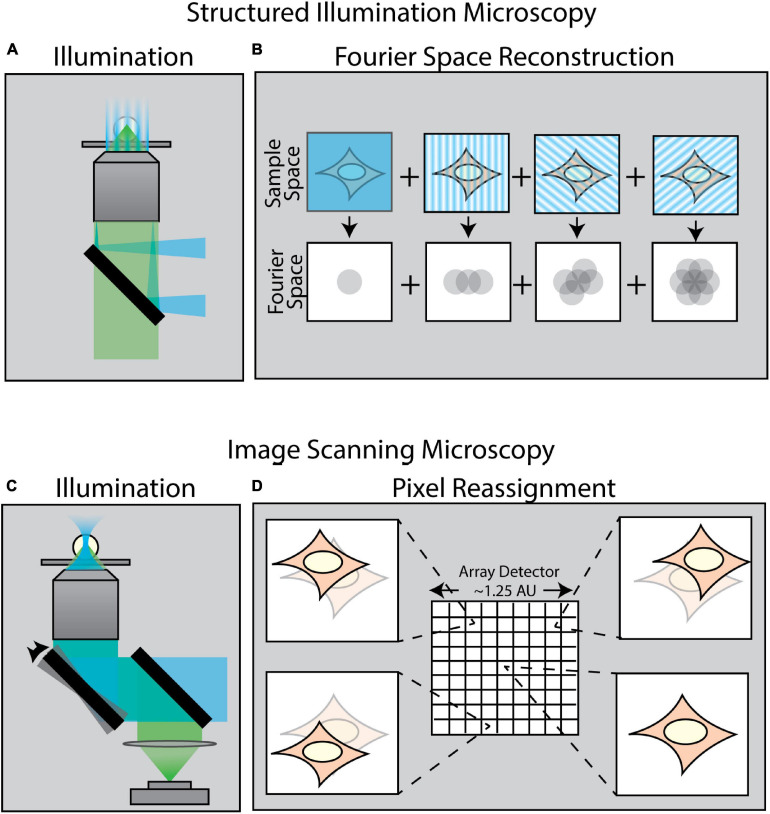
Methods of enhancing resolution in optical microscopy. **(A)** Structured Illumination Microscopy (SIM) is a widefield technique capable of up to twofold resolution enhancement in all directions. It relies on projecting a sinusoidal illumination pattern onto the sample to generate Moiré interference. **(B)** The illumination pattern is rotated and shifted over a series of 9–15 images to introduce extended frequency information into the Fourier space of the image. **(C)** Image Scanning Microscopy (ISM) uses an illumination scheme identical to laser-scanning confocal microscopy; however, the scanned image is collected by an array detector in which each pixel in the array acts as an individual pinhole-restricted point detector. **(D)** In contrast to confocal microscopy wherein a single image is formed via a single point detector, each pixel in an ISM array detector captures its own unique image of the sample from a slightly different angle. This information is computationally combined to create an enhanced resolution image.

A variety of techniques in recent years have been developed to surpass the diffraction limit. One class of these methods is collectively known as enhanced resolution techniques (Figure 2). Such techniques provide a maximum twofold improvement in spatial resolution in all directions, but are still ultimately bound by diffraction. Examples of such enhanced resolution methods include Structured Illumination Microscopy (SIM) (Figures 2A,B; Gustafsson, 2000; Gustafsson et al., 2008) and Image Scanning Microscopy (ISM) (Figures 2C,D; Sheppard, 1988; Müller and Enderlein, 2010; Sheppard et al., 2013) – of which the most popularized commercial system is the Zeiss Airyscan module (Huff, 2015). These methods are particularly well-suited for force measurements as they are relatively fast and are, in general, compatible with live samples.

**FIGURE 3:**
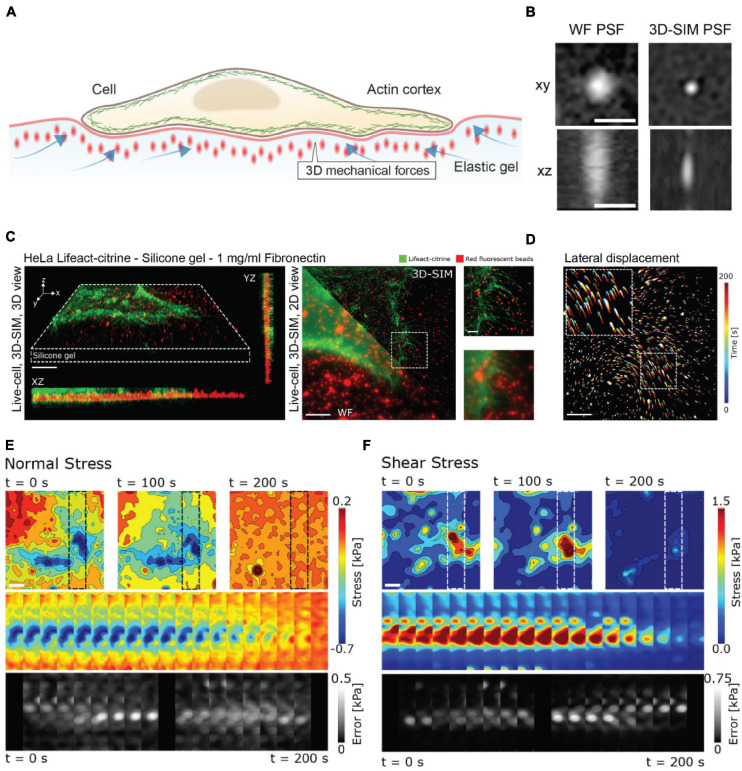
3D-SIM improves resolution and precision in measuring lateral and axial forces in TFM. **(A)** Schematic representation of 3D-TFM. **(B)** A comparison of PSFs demonstrates the enhanced resolution of 3D-SIM as compared to widefield (WF) microscopy. Scale bar: 0.5 μm. **(C)** (Left) Volumetric rendering and axial projections of 3D-SIM images of a HeLa cell expressing Lifeact-citrine (green) on a TFM substrate with fluorescent fiducial markers (red). (Right) A comparison of WF and 3D-SIM highlights the enhanced resolution. The dashed box inset for both WF and 3D-SIM is shown for direct comparison. Scale bar: 5 μm. **(D)** 2D translation of fiducial markers is shown color-coded for time, demonstrating dynamic cell-generated forces. Scale bar: 5 μm. **(E)** Normal and **(F)** shear stress fields from a 3D-SIM-TFM experiment. Top panels show the stress fields at different points in time. Middle panels show the full time series of stress maps for the region of interest shown in the upper panels indicated by dashed boxes. The lower panels show the estimated error for each time point. Scale bar: 5 μm. Images are reproduced with permission from Colin-York et al. (2019).

As an example of the power of coupling enhanced resolution microscopy with force measurements, let us consider the recent work by Colin-York et al. (2019). Recognizing that many cellular processes occur on length scales below the diffraction limit, Colin-York et al. sought to improve the accuracy and resolution of TFM in all three directions with enhanced resolution microscopy (Figure 3A). The method of choice in this instance was 3D-SIM, as it allows for rapid, multicolor 3D imaging (11 ms per frame, 15 frames per SIM image plane). In TFM, the density of the beads in the substrate dictates the sensitivity of the force measurement (Colin-York and Fritzsche, 2018). In practice, the maximum bead density that can be incorporated into a traction force measurement is fundamentally dictated by the resolving power of the microscope, as higher resolution is needed to distinguish neighboring beads at a higher density. In studying the force generated during cell adhesion (Figures 3C,D), 3D-SIM-TFM enhanced the accuracy of measuring the normal and shear stresses over time (Figures 3E,F). The application of 3D-SIM was particularly necessary to more accurately determine the stresses perpendicular to the substrate, which are nominally much smaller than the shear stresses measured in 2D-TFM (Colin-York et al., 2019). Similar performance has recently been achieved through the incorporation of astigmatism in conjunction with TFM (Li et al., 2021). Astigmatism induces a shape change in the PSF that depends on axial position (Kao and Verkman, 1994; Holtzer et al., 2007). This optical distortion allows for high-precision determination of forces perpendicular to the substrate. Measuring these axial forces is important for revealing non-canonical mechanisms of cell motility (Legant et al., 2013). Though not quantified in the study shown in Figure 3, 3D-SIM-TFM is well-positioned to improve mechanistic insights with its unique capability of linking, with high resolution, biomechanical forces with morphological changes in the actomyosin network.

However, SIM and ISM can only improve the spatial resolution of a microscope by at most a factor of two. If additional resolving power is necessary, users may turn to a class of techniques collectively known as super-resolution microscopy. Super-resolution microscopy comes in a variety of adaptations, each designed to transcend the diffraction limit and achieve resolutions on the scale of tens of nanometers. One such adaptation is single molecule localization microscopy (SMLM) (Sauer and Heilemann, 2017). SMLM techniques, such as PALM (Betzig et al., 2006) and STORM (Rust et al., 2006), repeatedly image photo-switchable fluorophores to reconstruct biological features with near molecular-scale precision. The computational methods associated with localization-based microscopy have been leveraged to track particle deformations, for example in the context of phagocytosis (Vorselen et al., 2020), and recent strides have been made in coupling SMLM with DNA-based molecular force sensors (Brockman et al., 2020; Schlichthaerle et al., 2021). However, SMLM is still predominantly restricted to fixed samples, rendering them ineffective for studying dynamic force application.

On the other hand, stimulated emission depletion (STED) microscopy (Hell and Wichmann, 1994) is a super-resolution method that is compatible with dynamic force measurements. By leveraging the photophysics of fluorophores and altering the traditional illumination schemes, STED microscopy can reach resolutions beyond SIM and ISM. However, due to the relatively high light dose, STED microscopy provides only a brief window of opportunity to study dynamic forces before incurring photodamage. Despite this limitation, STED microscopy has been combined with TFM (Colin-York et al., 2016). This allowed for a fivefold increase in the density of the bead field relative to diffraction-limited methods, improving the sensitivity of the associated traction force measurements. This came at the cost of severely limited imaging duration, as compared to 3D-SIM-TFM. Such compromise is essential for mechanobiological studies because photodamage can lead to significant alterations in both morphology and function, ultimately leading to apoptosis in some cases (Icha et al., 2017). In measuring forces, such light-induced phenomena cannot be overlooked.

### Photodamage

The act of illuminating the sample for imaging will inherently lead to photodamage to some extent. As a result, balancing the inevitable photodamage with the required imaging parameters will always be a necessary compromise. Photodamage manifests itself in two primary forms – photobleaching and phototoxicity – which can affect force measurements in distinct ways. Photobleaching (Diaspro et al., 2006b; Waters, 2009) causes a decrease in fluorescence signal-to-noise ratio (SNR), which subsequently reduces contrast and sets the photon budget of an experiment (Wait et al., 2020). This can in turn severely compromise either the timescale of forces that can be measured or the length of time one may observe and perform measurements. Equally important, the poor SNR caused by photobleaching can significantly increases the error in force measurements. On the other hand, phototoxicity results from light-induced damage to a live specimen. The unchecked damage to specimen health and viability calls into question the physiological relevance of the entire force measurement assay. Unfortunately, assessing phototoxicity is often not trivial. In practice, the specimen health is routinely assessed by empirical observation of morphology, which is not always a reliable phototoxicity metric (Icha et al., 2017). However, there are now several studies that explore more robust, quantitative, and sample-specific methods that focus on biological functions over time (such as cell division and proliferation) at various doses of light (Tinevez et al., 2012; Douthwright and Sluder, 2017; Laissue et al., 2017; Tosheva et al., 2020).

In principle, photodamage can be alleviated by lessening the light dose on the sample (Icha et al., 2017). Unfortunately, many widely used imaging methods are not fundamentally designed to make lowering the light dose their primary operational principle (Tinevez et al., 2012); in fact, several of these common imaging techniques are particularly prone to incurring photodamage. Confocal microscopy, for example, often induces photodamage for two reasons. First, the light intensity at the focal plane in confocal microscopy is usually in the range of 10^2^–10^6^ W/cm^2^ (Pawley, 2006; Ettinger and Wittmann, 2014), essentially exposing the biospecimen to 10^3^–10^7^ fold higher irradiance than lifeforms on earth have evolved to withstand (Seidlitz et al., 2001; Chen et al., 2014). Second, the excitation light illuminates the sample both above and below the observational plane. The confocal pinhole merely serves to block out-of-focus *emitted* light and does not prevent the excessive excitation light from damaging the sample outside of the focal plane. One means of mitigating such excessive and unnecessary illumination is to restrict the excitation light to the imaging plane. This can be achieved in several ways.

One method to confine the excitation light is total internal reflection fluorescence (TIRF) microscopy (Axelrod, 1989). TIRF operates by introducing light at or above the critical angle to prevent its propagation into the sample. When this occurs, only fluorophores at the cell-substrate interface are excited. The advantages of using TIRF microscopy are twofold. First, it reduces photodamage by limiting the excitation of the sample solely to within a few hundred nanometers of the coverslip. Second, this restricted excitation plane leads to a higher signal-to-background ratio because unnecessary excitation of fluorescent molecules beyond the focal plane is significantly minimized. This improved contrast allows users to lower the overall intensity of the excitation source. Together, these combined benefits make TIRF microscopy an ideal technique for minimizing photodamage. There are several canonical uses of TIRF microscopy with force measurements, primarily with TFM and FRET tension sensors. For example, coupling TIRF microscopy with FRET tension sensors has been used to study the distribution of forces generated by single integrins (Morimatsu et al., 2013). In addition, the reduced photobleaching associated with TIRF microscopy is particularly useful in FRET applications as unequal photobleaching rates between the donor and acceptor fluorophores can skew ratiometric calculations over time. In a similar way, TIRF microscopy facilitates correlating TFM with biological structures through reduced background (Gutierrez et al., 2011). Coupling of TIRF and TFM [and recently TIRF, SIM, and TFM (Barbieri et al., 2021)] has been used extensively to improve biological force measurements, demonstrating for example the colocalization of nascent focal adhesions with traction stresses (Han et al., 2015). Unfortunately, the specificity of TIRF illumination to the sample-coverslip interface precludes its use when the forces of interest have to be measured at a deeper plane away from the coverslip.

**FIGURE 4:**
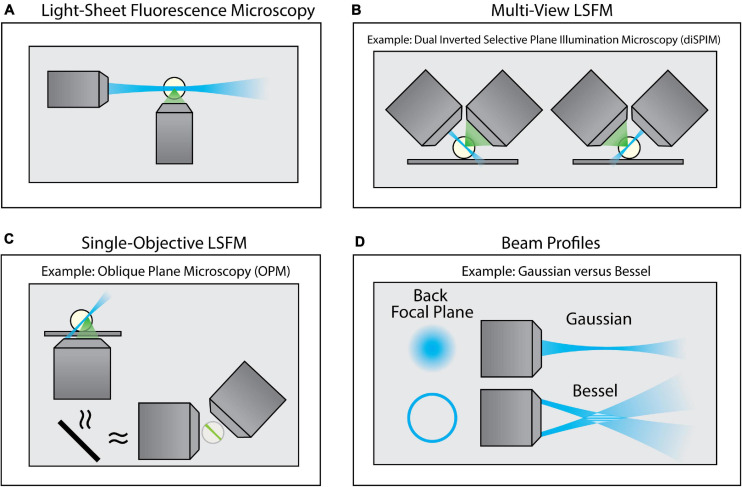
Variants of Light-Sheet Fluorescence Microscopy (LSFM). **(A)** Using an illumination objective lens, conventional LSFM illuminates a thin section of the sample that is coplanar with a separate detection lens. This restricts excitation to only the portion of the sample being imaged, thus improving contrast and minimizing photodamage. **(B)** Multi-view LSFM (e.g., diSPIM) can achieve multiple viewing angles by alternating the function of both objective lenses (as shown) or by incorporating multiple detection objective lenses to image the sample. Computational fusion of these multiple viewing angles can enable isotropic resolution, as well as mitigate attenuation artifacts. **(C)** Single-Objective LSFM (e.g., OPM) uses the same objective lens for both excitation and detection, providing adaptability for a wide range of samples and external devices. **(D)** Light sheets can be formed with different beam profiles (e.g., Gaussian or Bessel beams) that offer unique advantages for specific biological applications.

To overcome this limitation, a class of imaging techniques known either as light-sheet fluorescence microscopy (LSFM) or selective plane illumination microscopy (SPIM) can be used (Figure 4). These methods introduce a thin sheet of excitation light across the specimen that is coplanar with the image plane (Figure 4A). By sweeping the light sheet through the sample, LSFM can provide optical sectioning and contrast comparable to TIRF microscopy, but throughout the entire depth of the specimen. There exists a breadth of LSFM implementations, including multi-view LSFM (Figure 4B; Tomer et al., 2012; Kumar et al., 2014) and single-objective LSFM (Figure 4C; Bouchard et al., 2015; Liu et al., 2019; Yang et al., 2019; Sapoznik et al., 2020). Additionally, different light sheet profiles are available (Figure 4D; Durnin et al., 1987, 1988; Huisken et al., 2004; Planchon et al., 2011; Chen et al., 2014), each with their own specific benefits and limitations. Furthermore, commercialization of LSFM both through standalone systems as well as add-on LSFM modules that can be merged with conventional inverted microscopes (Fadero et al., 2018) has increased the accessibility of this method. A full survey of LSFM methodologies is beyond the scope of this Review; readers are encouraged to refer to several excellent reviews of this class of microscopes (Santi, 2011; Reynaud et al., 2014; Stelzer, 2014; Girkin and Carvalho, 2018).

**FIGURE 5:**
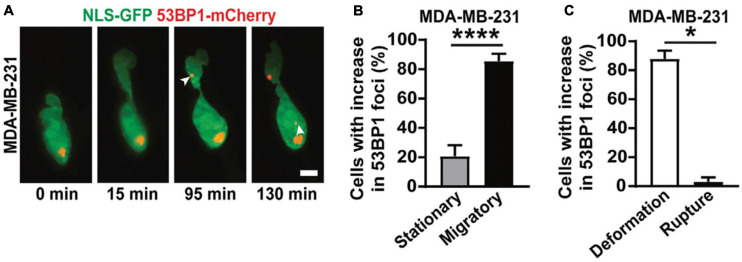
3D LSFM combined with confined migration minimizes photodamage during force-based experiments. **(A)** LSFM images of an MDA-MB-231 cell expressing GFP-tagged nuclear localization sequence (NLS-GFP), in green, and DNA damage marker (53BP1-mCherry), in red, navigating a 3D collagen matrix. The white arrows indicate new sites of DNA damage. Scale bar: 5 μm. **(B)** The percentage of MDA-MB-231 nuclei that show new sites of DNA damage when stationary or migratory. **** represents *p* < 0.0001 for a Fisher’s test. **(C)** The percentage of MDA-MB-231 nuclei that show DNA damage due to deformation alone or due to nuclear rupture. * represents *p* < 0.05 for a chi-square test. Images are reproduced with permission from Shah et al. (2021).

With the increasingly widespread adoption of LSFM, the benefits of minimizing light exposure have likewise been leveraged in 3D biomechanical force studies. One such example is the recent work of Shah et al. (2021). During tumor invasion and metastasis, cells encounter interstitial spaces that force the cell – and more specifically, the nucleus – to undergo drastic deformations. This compression can often lead to nuclear rupture and DNA damage (Denais et al., 2016). To study this confined migration process and the associated DNA damage, LSFM was used to follow cells as they navigated through narrow pores within 3D collagen matrices (Figure 5A). The unique capabilities of LSFM enabled imaging of multiple color channels in 3D over the course of several hours. Laser-scanning confocal microscopy, however, impeded similar experiments by restricting the image to only a single plane (2D) due to photodamage. 3D LSFM imaging was essential for unambiguously detecting formation of new DNA damage foci for two reasons. First, confusion occurs when imaging this process in 2D, as the appearance of foci in this case can be attributed either to a new breakage in DNA or an existing damage site coming into focus. 3D imaging disentangles these two scenarios. Second, it is well documented that over-exposure of light itself can induce DNA damage (Sinha and Häder, 2002), which can conflate conclusion about the role of deformation. The reduced light exposure with LSFM helped isolate the mechanism by which damage sites were produced. Together, these benefits lent to the conclusion that nuclear deformation alone, independent of nuclear rupture, is sufficient to increase double-stranded DNA breaks (Figures 5B,C). While the present study did not quantify the magnitude of the forces that these cells underwent during confined migration, coupling of LSFM with labeled matrices would allow future investigators to quantify both the forces applied to the cell by the matrix, as well as the forces that the cell applies to generate motion. Such studies could then infer the magnitude of forces necessary to induce DNA damage or the mechanisms of force generation to facilitate movement through narrow constrictions. The significant reduction in photodamage offered by LSFM is an important technical advance, allowing biologists to probe the roles of biomechanical forces during morphogenesis (Bambardekar et al., 2015; Vedula et al., 2017; de Medeiros et al., 2020), phagocytosis (Nelsen et al., 2020; Vorselen et al., 2021) and T cell engagement (Tamzalit et al., 2019). In general, the ability to observe biological specimens under relatively low-stress conditions has opened new avenues for biologists to pursue their questions within a more physiologically relevant context while pushing the previous limitations on speed, dimensionality, and depth.

### Speed and Dimensionality

Studying complex biological processes often dictates 3D observations in multiple color channels, while maintaining sufficient temporal resolution, so that the dynamic interplay among key molecular players can be visualized. Such demanding, multi-dimensional experiments are essential for exploring a range of biological length scales, from single cells to whole organisms. In practice, however, greater image size and dimensionality will come at the cost of reduced imaging speed. Acquisition speed is a critical imaging parameter, as one needs to accurately follow in time the forces being studied. For certain physiological processes, the timescales are long enough such that the trade-off between speed and dimensionality is acceptable given conventional imaging methods. However, there are biological events that occur on single-second timescales – such as membrane tether rupture (Schmitz et al., 2008) and photoablation-induced tension relaxation (Kumar et al., 2006; Zhang et al., 2020) – that are best addressed by more advanced imaging methods.

**FIGURE 6:**
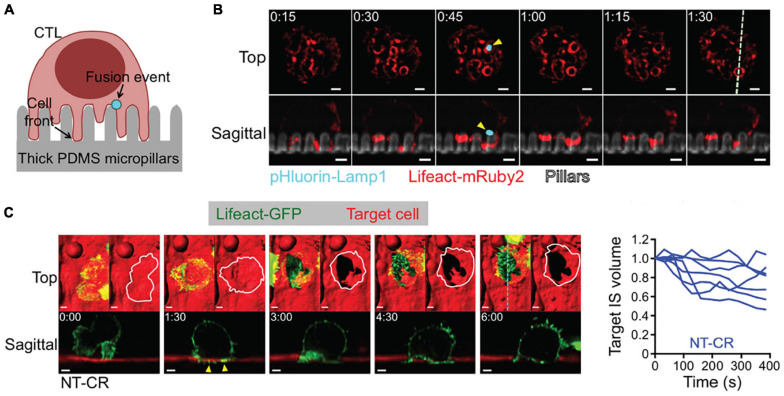
3D LSFM coupled with micropillar arrays improves temporal sampling during force measurements. **(A)** Schematic representation CTL engagement with micropillar arrays and the formation of synaptic protrusion and lytic granule fusion events. **(B)** LLSM of a CTL expressing Lifeact-mRuby2 (red) and pHluorin-Lamp1 (blue) engaged with a micropillar array (gray). The top row provides an x-y view from above. The bottom row shows an axial cross section of the region denoted by the dashed line in the upper right panel. Yellow arrows indicate the site of a fusion event. Time is given in minutes:seconds, scale bars: 2 μm. **(C)** (Left) LLSM of a CTL expressing Lifeact-GFP (green) engaging with a target cell (red). The top row provides an x-y view from above with a surface rendering visualization. The bottom row shows an axial cross section of the region denoted by the cyan dashed line in the upper right panel. Yellow arrows indicate protrusions into the target cell. Time is given in minutes:seconds, scale bars: 2 μm. (Right) Target cell volume plotted as a function of time. Each curve represents an individual CTL-target cell engagement. Images are reproduced with permission from Tamzalit et al. (2019).

By design, the versatility and gentle illumination of LSFM can be leveraged to tackle these demanding mechanobiological phenomena. As previously mentioned, LSFM minimizes out-of-plane fluorescence by restricting the excitation light to a thin sheet, thereby greatly improves image contrast. In comparison to widefield microscopy, this considerable contrast improvement enables shorter exposure times and faster imaging rates. While laser-scanning confocal microscopy can provide similar contrast to LSFM, it fails to offer the high imaging rates of LSFM. Together, the benefits of LSFM permit multi-channel volumetric acquisitions of single cells with rates approaching 1–5 s per volume. The work of Tamzalit et al. (2019) is an excellent case study on the benefits of improving speed and dimensionality in force measurements. The investigators sought to measure the forces associated with cytotoxic T lymphocyte (CTL) engagement with micropillar arrays (Figure 6A) and dynamically characterize the formation of synaptic protrusions. This experiment required multiple channels to visualize the micropillars themselves as well as track cellular structures associated with immune synapse formation. The investigators used lattice light-sheet microscopy (LLSM) (Chen et al., 2014), as it is particularly well-suited for fast 3D sub-cellular imaging. This coupling of LSFM with micropillar arrays gave sufficient temporal resolution to visualize actin protrusions permeating the array of micropillars and localize lytic granule fusion sites during synapse formation (Figure 6B). Furthermore, LSFM was used to monitor CTL-induced deformation of target cells, leading to quantification of synapse volume as a function of time (Figure 6C). Intriguingly, the investigators successfully used 2D confocal microscopy to track micropillar flexure, but were unable to achieve the necessary temporal resolution in 3D to monitor synapse formation (unpublished data). This lays the groundwork for using LSFM to quantify in 3D the full bending, twisting, and translation of micropillar arrays, rather than only the conventional 2D translations. This added dimensionality can be used to extract axial forces and to determine the precise location of force generation.

**FIGURE 7:**
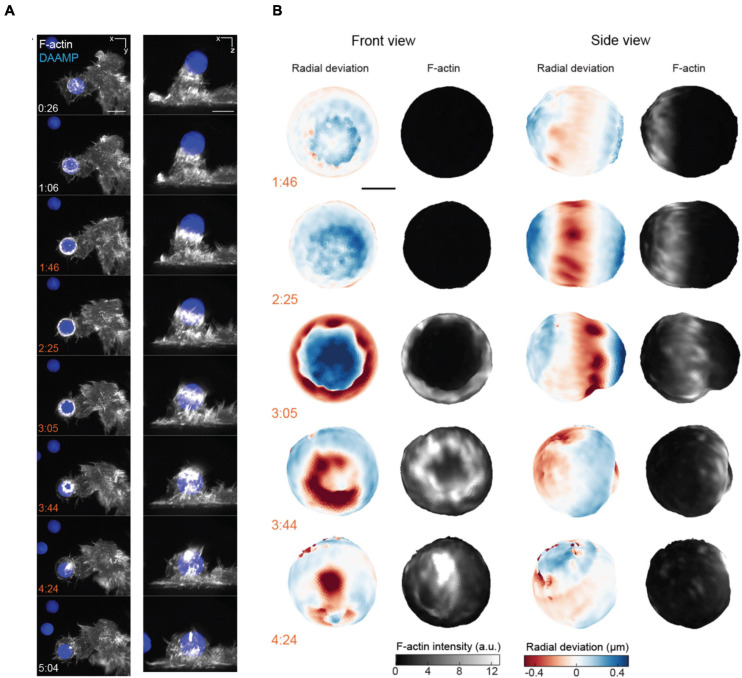
3D LSFM combined with microparticle traction force microscopy monitors target deformation during phagocytosis. **(A)** Maximum intensity projections of RAW macrophages expressing Lifeact-mEmerald (white) engulfing deformable particles labeled with AlexaFluor 647 (blue) imaged with LLSM. Time is given in minutes:seconds, scale bar: 5 μm. **(B)** Front and side view projections of target deformations and actin intensity around the target particle at various time points during the engulfment process. Time denoted in minutes:seconds, scale bar: 3 μm. Images are reproduced with permission from Vorselen et al. (2021).

Recently, investigators have coupled AFM with LSFM (AFM-LS) to either directly image the plane of applied force with up to 10 ms temporal resolution (Beicker et al., 2018), or collect multi-channel volumetric images with simultaneous correlated force measurements (Nelsen et al., 2020). The additional speed and dimensionality that AFM-LS provides allowed investigators to separate the roles of individual nuclear substructures in response to an applied force (Hobson et al., 2020) as well as correlate actin dynamics with engulfment forces during phagocytosis (Nelsen et al., 2020). Similarly, LSFM was coupled with microparticle traction force microscopy to both quantify the forces associated with phagocytosis and identify novel actin structures responsible for their generation (Figures 7A,B; Vorselen et al., 2021). These examples display how microscopy can provide additional insight into the cellular structures responsible for generating or responding to mechanical stimuli. Furthermore, researchers have combined FRET with the benefits of LSFM, and developed publicly available software to analyze this challenging type of data (O’Shaughnessy et al., 2019). This opens the door for future studies to utilize FRET for quantifying molecular-scale tension forces in 3D at unprecedented speeds. These examples highlight how LSFM, in conjunction with force measurement, unlocks information that traditional microscopy cannot provide. We have thus far dwelled on mechanistic studies at the cellular level; yet, biomechanical force is an indispensable signal and regulator in morphogenesis and development as well (Guillot and Lecuit, 2013; Heisenberg and Bellaïche, 2013; Agarwal and Zaidel-Bar, 2021). When force measurements must be performed in the physiological context of a whole organism, the tissue heterogeneity, light scattering, and large-scale specimen movement can easily affect the precision and outcome. Overcoming these challenges will require further technical advances.

### Imaging Depth

Imaging whole tissues and embryos presents a gamut of new challenges as compared to imaging single cells. While techniques such as LSFM have begun to enable biomechanical force measurements during tissue morphogenesis (Bambardekar et al., 2015; Vedula et al., 2017; de Medeiros et al., 2020), heterogeneity in large samples can cause significant aberrations due to refraction, scattering, and absorption (Schwertner et al., 2007). These complications will reduce spatial resolution and contrast, rendering force measurement experiments significantly less accurate or even impossible.

**FIGURE 8:**
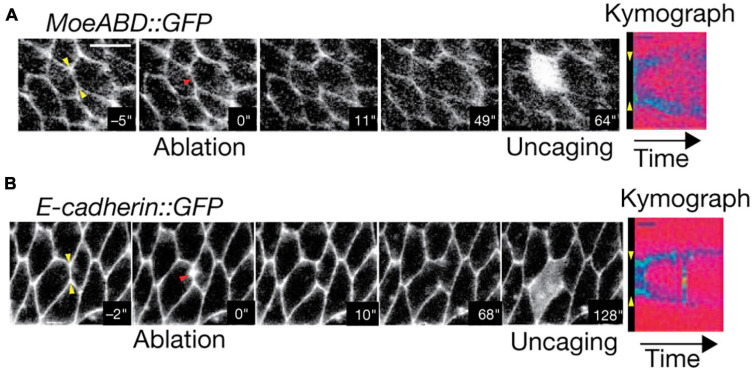
Two-Photon photoablation reveals mechanical tension *in vivo*. Image sequences of *Drosophila* embryo expressing **(A)** MoeABD-GFP and **(B)** E-cadherin-GFP during 2P photoablation of cell-cell junctions. The red arrow in the second panel from the left represents the site of photoablation. Kymographs at the right (taken at the yellow arrows shown in first panel) show the release and retraction of the ablated region. Uncaging of fluorescein with three-photon microscopy (panel 5) demonstrates that the cell integrity is not disrupted by the photoablation. Scale bar: 5 μm. Images are reproduced with permission from Rauzi et al. (2008).

To gain better light penetration depth when imaging large samples, one may turn to using two-photon (2P) excitation (Denk et al., 1990; Diaspro et al., 2006a). In contrast to conventional fluorescence, 2P excitation uses two photons of double the required wavelength to excite a fluorophore. This permits deeper imaging for two reasons. First, longer wavelength excitation will generally experience fewer interactions with the sample. Second, 2P excitation events are far more rare than traditional fluorescence (Denk, 1996; Denk and Svoboda, 1997; Svoboda and Yasuda, 2006); therefore, emission occurs within a much smaller excitation volume. This minimizes background fluorescence, particularly in deep tissue. 2P excitation light is typically raster-scanned across the sample, similar to confocal microscopy. However, it has also been implemented in LSFM configurations to combine the benefits of both techniques (Truong et al., 2011; Mahou et al., 2014; Wolf et al., 2015). The predominant use of 2P microscopy in measuring biomechanical forces is through photoablation and measurement of subsequent tension relaxation (Shen et al., 2005; Rauzi et al., 2008, 2010; Ratheesh et al., 2012; Michael et al., 2016; Yamaguchi et al., 2020). While many studies leverage ultra-violet pulsed lasers to ablate specimen targets (Kiehart et al., 2000; Hutson et al., 2003; Fernandez-Gonzalez et al., 2009; Colombelli and Solon, 2013; Smutny et al., 2015; Hara et al., 2016; Zhang et al., 2020), the use of 2P microscopy improves both ablation depth and precision. This is of particular importance when measuring tension *in vivo*, as is exemplified by the work of Rauzi et al. (2008). In this case, 2P microscopy permitted ablation of individual *Drosophila* embryo cell-cell junctions during cell intercalation without disrupting the plasma membrane (Figures 8A,B). This led to the observation that tension is anisotropic within the tissue, which was posited as a mechanism to promote tissue elongation (Rauzi et al., 2008). However, 2P microscopy has rarely been used in conjunction with other force measurement techniques outside of photoablation. This is primarily due to the relative scarcity of force probes that are specifically designed for whole tissues. 2P microscopy furthermore has a limited repertoire for biomechanical studies in deep tissue: its acquisition speed limited by raster-scanning and its multicolor capability complicated by large excitation overlap between fluorophores.

To push the multidimensional capability deeper into the context of a whole organism, approaches based on adaptive optics (AO) have been developed. The overall goal of AO is to measure the image distortion induced by the sample and use that information to counter the aberration, rendering crisper and brighter images. One such method – the “guide star” technique (Primmerman et al., 1991) – images point sources at various locations in a sample to characterize the local wavefront distortion. An adaptive element, such as a deformable mirror, then applies the inverse of that distortion to recover the un-aberrated image. AO can be integrated into both standard microscopes (Azucena et al., 2011; Tao et al., 2011) as well as more advanced systems, such as the lattice light-sheet microscope (Liu et al., 2018). Likewise, AO can also be used to correct the excitation light wavefront, which is particularly important for methods that use some form of spatially structured excitation. The next frontier of exploration into the complex physiology of biomechanical force transduction will demand the strategic integration of (i) purpose-designed *in vivo* force sensors, (ii) advanced optical microscopes, and (iii) computational tools.

## Data Analysis and Handling

Any microscopy-based force measurement technique will require image processing and analysis to achieve meaningful results. Two of the most common techniques are particularly dependent in this regard: (i) traction force microscopy (TFM) and (ii) FRET tension biosensors. Both techniques require careful and often complex image-based calculations to arrive at an accurate force measurement. Here, we discuss the data handling and analysis challenges of these two methods, particularly in the context of their implementation with advanced imaging technologies.

### Analysis of Traction Force Microscopy Data

Measuring the displacement of embedded fiducial markers provides a quantitative view into the minute forces cells exert on their environment. As described previously, TFM is considered one of the “classical” techniques of force measurement in mechanobiology (Harris et al., 1980; Dembo and Wang, 1999). The concept of TFM is deceptively simple: optical tracking of fiducial markers provides data that can be mathematically related to the physical forces that cells exert on a substrate. However, this straightforward premise requires several complex decisions in experimental design, analysis, and optical configuration to achieve the most biologically relevant data.

First, the choice for fiducial markers is critical to data quality. To maximize the force sampling density, it is recommended that fiducials be smaller than the spatial resolution of the optical system (e.g., 100 nm diameter). While larger particles (e.g., 500 nm diameter) can be more easily localized in lower resolution optical instruments, it restricts the maximum fiducial density in the substrate. This is important because higher density will provide better sampling of the minute changes in the displacement field, which translates to more refined force calculations. The maximum density of fiducials, and therefore the ability to sample biomechanical force, is ultimately limited by the optical resolution. However, two methods can circumvent this barrier. First, beads of multiple colors can be placed within the same substrate and imaged separately (Gardel et al., 2008; Sabass et al., 2008; Plotnikov et al., 2012). This technique, referred to as “high resolution TFM,” improves the force measurement sampling by several fold (Plotnikov et al., 2014), but requires accurate multi-channel alignment. Recent advances in optical microscopy, such as SIM and ISM, enable similar gains in fiducial density by enhancing the spatial resolution up to twice the diffraction limit (Colin-York and Fritzsche, 2018; Colin-York et al., 2019; Barbieri et al., 2021). A combination of both enhanced-resolution microscopy and multi-color fiducial markers would allow even higher fiducial densities beyond either method alone. Fiducial density aside, it is also important to consider the assumptions inherent to TFM analysis.

There are two important assumptions that must be made for later force calculations. First, it is presumed that any motion of the fiducial markers is due only to cell-generated forces. This assumption does not always hold true; drift or degradation of the substrate itself can lead to non-biological fiducial displacement. Such effects can be identified either by imaging substrates without cells attached or imaging a large enough field of view to capture non-perturbed regions for comparison. The second potentially erroneous assumption is in the mechanical uniformity of the substrate. Numerous substrates have been used to interrogate specific behaviors, from simple polyacrylamide gels (PAAG) to more complex patterned microsurfaces (Balaban et al., 2001; Beningo et al., 2001; Tamzalit et al., 2019). To properly calculate force, the analysis must assume that the material rigidity is constant across the whole volume. In other words, TFM relies on the presumption that every bead in the substrate will experience an identical displacement from a given amount of force. Mechanical homogeneity of the substrate can be assessed by conventional stiffness measurement assays such as atomic force microscopy (Tse and Engler, 2010).

For conventional TFM, individual beads are optically tracked using localization or correlation-based particle image velocimetry (Butler et al., 2002; Toli-Nørrelykke et al., 2002). This analysis produces a set of bead displacements over time. From there, the mathematical and physical relationships that connect these displacements to underlying forces must be applied properly (Huang et al., 2019). These intensive calculations compound any error in the initial optical measurement of the displacement field. The use of optical-sectioning methods such as TIRF and LSFM minimizes inaccuracies by dramatically increasing the fiducial signal contrast and therefore localization precision (Gutierrez et al., 2011; Han et al., 2015; Barbieri et al., 2021). Finite element analysis, Bayesian models, or various regularization methods can provide more sophisticated information to better represent the true nature of complex biomechanical systems. It is beyond the scope of this review to compare mathematical models used in TFM which have been well-covered in the literature (Yang et al., 2006; Zielinski et al., 2013; Kulkarni et al., 2018; Huang et al., 2019). Better models and advanced instrumentation are necessary to investigate more complex multi-cellular systems (Franck et al., 2011; Tang et al., 2014), which require considering both cell-substrate and cell-cell interactions. While TFM represents a powerful technique to measure nanoscale forces exerted by various biological systems, it lacks a means to identify the source(s) of such forces with molecular specificity.

### Analysis of FRET Tension Biosensor Data

Genetically encoded FRET-based biosensors can quantify biological tension with molecular specificity and picoNewton sensitivity (Cost et al., 2015, 2019; LaCroix et al., 2015; Gayrard and Borghi, 2016; Gates et al., 2019). This technique relies on microscopy to measure the sensor FRET efficiency, or the degree of energy transfer occurring between the donor and acceptor fluorophores to infer a tension force. However, analysis of such data must be performed judiciously, and advanced imaging methods can both complicate and ameliorate the generation of meaningful biological conclusion.

Arguably the most common way to measure FRET efficiency requires computing the ratio of acceptor-to-donor fluorescence intensity (Hoppe, 2007). However, numerous factors can render a naïvely calculated ratiometric image utterly non-informative. First, cross-excitation and spectral overlap will produce erroneous signal in both donor and acceptor channels. To account for this, it is imperative to prepare proper control samples labeled with tension sensors containing only donor or acceptor fluorophores alone (O’Shaughnessy et al., 2019).

However, other factors will also affect the accuracy of ratiometric FRET calculations. Microscope illumination intensity may vary by a surprising amount across the field of view. To account for this variability, homogeneous fluorescent samples should be imaged in both donor and acceptor channels (Model and Burkhardt, 2001) as a reference. Such “shade correction” can be of particular importance in more advanced microscopes such as LSFM or other patterned illumination systems that can be especially prone to uneven excitation light. In addition, the accuracy of ratiometric FRET measurements is dependent on the precision of color channel registration. Enhanced resolution techniques such as SIM and ISM can often reveal misalignments that are not apparent with lower resolution techniques (Baddeley et al., 2011). Multicolor fiducial markers provide a way to computationally register color channels with sub-diffraction accuracy (Manders, 1997). Finally, it is also important to normalize the measured donor signal by the overall donor photobleaching rate within the cell (Zal and Gascoigne, 2004), although LSFM can often reduce photobleaching to near negligible levels (Chen et al., 2014). While complex, such analysis can be successfully implemented to create high-quality FRET images. For example, O’Shaughnessy et al. (2019) have developed freely available FRET analysis software that is well suited for dealing with large volume LSFM data.

Other advanced imaging techniques can simplify ratiometric FRET measurements and analysis. For example, spectral imaging permits detection and computational extraction of both donor and acceptor signals from a single acquisition (Ecker et al., 2004), making image registration and crosstalk correction unnecessary. However, spectrally resolved detection is most common in laser-scanning confocal microscopes (Zimmermann et al., 2003), with fewer implementations in other more advanced modalities. Additionally, the presence of endogenous fluorescent molecules in the sample can complicate accurate analysis (Rossetti et al., 2020). Once an accurate ratio of donor-to-acceptor fluorophore intensity has been established, an image of FRET efficiency can be calculated (Chen et al., 2006).

Conversely, fluorescence lifetime imaging microscopy (FLIM) is an alternative to intensity-based methods for measuring FRET efficiency (Becker, 2012; Ebrecht et al., 2014). In this case, the donor fluorescence lifetime, rather than the ratio of donor and acceptor intensities, can be used to determine FRET efficiency directly. FLIM is a powerful technique to characterize FRET-based tension sensors and is subject to far fewer confounding issues than ratiometric approaches. However, the necessary instrumentation is more specialized and less commonly available than other imaging technologies. It can also suffer from slower acquisition speed compared to widefield or raster-scanning techniques, although recent advancements have begun to address this issue. For example, Mizuno et al. (2021) devised a system whereby a sample was illuminated sinusoidally in time, with a unique modulation frequency at each pixel location. Through this, they were able to perform frequency domain FLIM without serially scanning a focal spot across the sample, thereby greatly improving FLIM speed over previous methods.

Regardless of the method chosen, an appropriate calibration curve is required to translate a measured FRET efficiency into force (Grashoff et al., 2010; Gayrard and Borghi, 2016). Such a relationship is essential to establish because FRET efficiency will not, in general, be linearly proportional to force. The most common procedure to experimentally calibrate FRET tension biosensors has been via the use of optical tweezers (Hohng et al., 2007; Grashoff et al., 2010). While this procedure can be technically challenging, a number of FRET-based tension sensors have been previously characterized in the literature (Grashoff et al., 2010; Ringer et al., 2017; Salmon and Bloom, 2017; Li et al., 2018), allowing subsequent users to more easily translate their own measured image data into high quality force maps.

## Discussion

Biomechanical forces underpin a wide array of life processes, ranging from mediating cellular behavior, regulating signaling pathways, sculpting morphogenesis, governing embryonic development, facilitating immune response, to influencing the pathogenesis of many diseases that include cancer, cardiovascular failure, and musculoskeletal disorders. It is therefore no surprise that mechanobiology continues to gain prominence and the attention of biologists across many fields. Unlike many biochemical signals, biomechanical forces cannot be directly tagged for visualization and therefore must be inferred. The methods discussed here provide innovative solutions for measuring force with exquisite sensitivity. However, in practice, their accuracy and precision are ultimately limited by the capabilities of the accompanying microscope. In that regard, many emerging microscopy techniques hold the promise of considerable technological improvements which, when appropriately integrated into a force measurement assay, can reveal further biomechanical insights that have hitherto remained out of reach.

One considerable barrier not commonly discussed in the surveys of emerging microscopy technologies is the lack of accessibility for most biologists to this cadre of instruments, many of which are not commercially available. These instruments are usually developed in engineering or biophysics laboratories that historically have limited interaction with biologists. The inherent academic compartmentalization between research disciplines as disparate as life sciences and optical engineering can often impede the adoption of the nascent imaging technologies by mechano-biologists. This is especially the case when the creation of new microscopy technology far outpaces the speed of commercialization. However, with the idea of open science continuing to gain prevalence, many initiatives have been created to tear down these barriers. There are now numerous international and regional initiatives dedicated to bridging the chasm between technology development and adoption, thus making advanced microscopes and the associated expertise accessible to the broader life sciences community. Some of these centers – e.g., the Advanced Imaging Center at HHMI Janelia Research Campus (Chew et al., 2017) and the Advanced Bioimaging Center at the University of California-Berkeley – are specifically designed to provide peer-reviewed, proposal-driven, free-of-charge accessibility to these emerging microscopy technologies well before they become commercially available. For initiatives such as these to make an effective impact, a myriad of institutional support and imaging science expertise is essential, requiring significant investment.

An alternative open-access model is the concept of a “traveling microscope.” One effort by Huisken and colleagues, dubbed the “Flamingo,” seeks to address instrument access by designing a modular, portable light sheet system (Power and Huisken, 2019). The instrument is customizable and shipped with full installation instructions to institutes across the world. While this concept is limited to optical techniques that will survive shipping, it can be a useful option for mechanobiologists who are impeded by instrument access through any other means. Another laudable approach is the recent paradigm shift toward sharing design blueprints of newly developed, pre-published microscopy tools, through open access mechanisms (Pitrone et al., 2013; Chew et al., 2017; Millett-Sikking et al., 2019; Voigt et al., 2019; Kumar et al., 2021). For groups with the necessary expertise and resources, replicating these instruments is a viable and sustainable choice.

Taken together, the remarkable confluence of advanced optics and the unprecedented access to microscopy resources makes this the opportune time to sharpen the toolkit for biomechanical force measurement. Advanced microscopy now enables imaging with unprecedented versatility; developing force probes that fully take advantage of such tools for *in vivo* mechanobiological studies is a necessary next step. Overall, synergistic development of force measurement techniques, new optical tools, and novel computational strategies will be critical for elevating biomechanical studies to the next frontier.

## Author Contributions

All authors contributed to the conceptualization, writing and editing of this manuscript, and approved the submitted version.

## Conflict of Interest

The authors declare that the research was conducted in the absence of any commercial or financial relationships that could be construed as a potential conflict of interest.

## Publisher’s Note

All claims expressed in this article are solely those of the authors and do not necessarily represent those of their affiliated organizations, or those of the publisher, the editors and the reviewers. Any product that may be evaluated in this article, or claim that may be made by its manufacturer, is not guaranteed or endorsed by the publisher.
